# A Study of the Avalanche Multiplication and Excess Noise in Al_*x*_In_1–*x*_As_γ_Sb_1‑__γ_ Avalanche Photodiodes Lattice-Matched to GaSb

**DOI:** 10.1021/acsphotonics.5c02166

**Published:** 2026-02-13

**Authors:** Xiao Jin, Wenguang Zhou, Yang Zhao, Qingyu Tian, Xin Yi, Xiaofeng Tao, Adam Craig, Mrudul Modak, Andrew Marshall, Yingqiang Xu, Guowei Wang, John P. R. David, Gerald S. Buller

**Affiliations:** † Department of Electronic and Electrical Engineering, 7315 University of Sheffield, Sheffield S1 3JD, U.K.; ‡ Key Laboratory of Optoelectronic Materials and Devices, Institute of Semiconductors, 71243 Chinese Academy of Sciences, Beijing 100083, China; § Center of Materials Science and Optoelectronics Engineering, University of Chinese Academy of Sciences, Beijing 100049, China; ∥ China Electric Power Research Institute, Beijing 102209, China; ⊥ Institute of Photonics and Quantum Sciences, School of Engineering and Physical Sciences, 3120 Heriot-Watt University, Edinburgh EH14 4AS, U.K.; # Department of Physics, University of Lancaster, Lancaster LA1 4WA, U.K.

**Keywords:** avalanche photodiodes, SACM APD, excess noise, impact ionization, photodiodes, Al_x_In_1−x_As_γ_Sb_1‑__γ_, avalanche multiplication, SWIR

## Abstract

High-sensitivity linear-mode avalanche photodiodes (APDs) that operate beyond 1.65 μm and up to 2 μm require a narrow bandgap that also gives rise to high dark currents, especially when subject to the large electric fields necessary for avalanche multiplication. This has led to increasing interest in separate absorption, charge, and multiplication (SACM) detectors where the narrow bandgap absorber has a low electric field and the wider bandgap multiplication region provides the gain. A systematic study of Al_0.7_In_0.3_As_0.31_Sb_0.69_ grown lattice-matched on GaSb as the multiplication layer has been undertaken on p–i–n structures varying in width from 0.1 to 1.5 μm and the ionization coefficients and excess noise extracted over a wide electric field range (195 kV/cm–830 kV/cm). When integrated with a lattice-matched Al_0.3_In_0.7_As_0.64_Sb_0.36_ absorption layer, such an SACM APD is found to demonstrate a quantum efficiency of 64% and 10% for the wavelengths of 1.55 and 2 μm, respectively, at punch-through, without any antireflection coating. The device shows a maximum avalanche gain of 197 with an excess noise of 3.1 at a gain of 10. Such APDs can be potentially used in a receiver for many photon-starved applications, including gas sensing and LiDAR.

## Graphical abstract

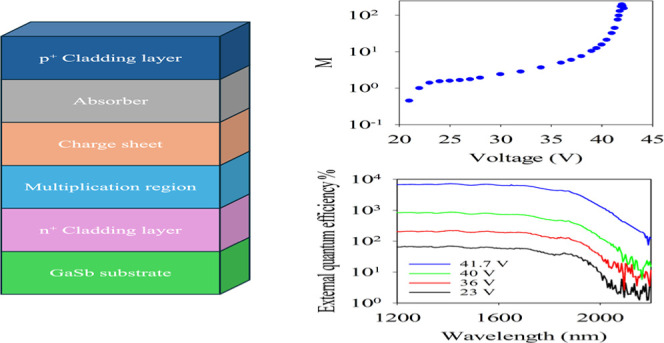

## Introduction

Avalanche photodiodes (APDs) operating in the short-wave infrared region (SWIR: 1.4–3 μm) have many civil and security applications, such as free space optical communication, remote sensing, and light detection and ranging (LiDAR).^1^ More recently, the demand for greenhouse gas monitoring, for example, the absorption band of CH_4_ (1.65 μm) and CO_2_ (2.05 μm), has led to the development of high-performance photodetectors operating beyond the cutoff of traditional InGaAs-based APDs. The signal-to-noise-ratio (SNR) in an APD-based receiver can be enhanced by utilizing the internal avalanche multiplication (*M*) to provide gain. However, this multiplication often comes at the cost of avalanche excess noise due to the stochastic nature of the impact ionization process, increasing the overall noise and eventually degrading the SNR.^2^ McIntyre’s local field theory defines this excess noise factor (*F*) as^3^

1 $$F\left(M\right)=kM+\left(1-k\right)\left(2-\frac{1}{M}\right)$$

where *k* = $\frac{\beta }{\alpha }$ (the ratio of hole ionization coefficient, β, and electron ionization coefficient, α, for electron-initiated multiplication.

This *F* value sets the limit of the maximum useful avalanche gain achievable for a given device, meaning that high-sensitivity APDs require a large SNR, which in turn necessitates the use of an avalanche material with a small *k* value.

InAs-^4^ and HgCdTe^5^-based APDs have been the main candidates for high-sensitivity light detection beyond 2 μm; unfortunately, due to their narrow bandgaps, they are generally operated at cryogenic temperatures to reduce device dark currents. Type-II superlattices (T2SL) of In_0.53_Ga_0.47_As and GaAs_0.51_Sb_0.49_ can be grown lattice-matched to the InP substrate with an effective bandgap that is smaller than either of these two materials, allowing photon absorption at wavelengths >2 μm but with the disadvantage of a reduced quantum efficiency.^6,7^ Combining a T2SL absorber with an InAlAs multiplication region has enabled detection out to 2.4 μm but with a relatively high *F*_e_ = 3.5 at *M*_e_ = 11.^8^ The advent of the Al_0.7_In_0.3_As_0.74_Sb_0.26_ ^9^ and wider bandgap Al_*x*_Ga_1–*x*_As_*y*_Sb_1–*y*_ alloy system lattice-matched to InP has enabled some impressively extremely low noise avalanche performance to be demonstrated, even at high gains.^10–17^ Nevertheless, this leaves us with the problem of not having a suitable lattice-matched absorber material to InP that can detect photons beyond 1.65 μm with a suitably high quantum efficiency.

Narrow bandgap alloys capable of detecting light beyond 1.65 μm such as InGaAsSb^18^ and Al_*x*_In_1–*x*_As_γ_Sb_1‑__γ_ ^19,20^ can be readily grown lattice-matched to GaSb. These can be integrated with a wide bandgap multiplication region in an SACM APD configuration to give rise to high-sensitivity detectors operating beyond the detection of conventional InGaAs-based APDs. Jin et al.^21^ recently showed that combining In_0.22_Ga_0.78_As_0.19_Sb_0.81_ with an Al_0.9_Ga_0.1_As_0.08_Sb_0.92_ multiplication region can give rise to an SACM APD that has a cutoff at 2.75 μm and demonstrates *F*_e_ = 4.5 at *M*_e_ = 20. However, most of the work in the literature on >1.65 mm APDs to date has focused on Al_*x*_In_1–*x*_As_*y*_Sb_1–*y*_-based SACM APDs lattice-matched to GaSb, where a lower aluminum composition alloy acts as the long-wavelength absorber and a higher aluminum composition acts as the low noise multiplication region.^22–25^ The initial low excess noise results in an Al_0.7_In_0.3_As_*y*_Sb_1–*y*_ p–i–n^26^ structure comparable to that of silicon were rapidly followed by an Al_0.4_In_0.6_As_*y*_Sb_1–*y*_/Al_0.7_In_0.3_As_*y*_Sb_1–*y*_ SACM APD structure capable of detecting just beyond 1.6 μm.^27^ Jones et al.^28^ then showed that p–i–n and SACM APD structures based on Al_0.7_In_0.3_As_*y*_Sb_1–*y*_ had a very low temperature coefficient of breakdown, and subsequently Yuan et al. showed from photomultiplication measurements that this material had a small β/α ratio.^29,30^ In 2020, Jones et al.^23^ showed that an EQE of 21% at 2 μm in an Al_0.3_In_0.7_As_*y*_Sb_1–*y*_/Al_0.7_In_0.3_As_*y*_Sb_1–*y*_ SACM APD at punch-through (*V*_pt_) could be obtained by reducing the aluminum in the absorber. Reducing the thickness of the narrow bandgap absorber to 200 nm reduced the dark currents by 2 orders of magnitude, and incorporating a photon trapping structure overcame the reduced absorption to give a QE of ∼22% at 2 μm.^31^ Lowering the aluminum in the absorber to 5% in an Al_0.05_In_0.95_As_*y*_Sb_1–*y*_/Al_0.7_In_0.3_As_*y*_Sb_1–*y*_ SACM APD further extended the absorption to 3.5 μm; however, the increased dark currents required the device to operate at 100 K.^32^

Growing Al_*x*_In_1–*x*_As_*y*_Sb_1–*y*_ as a random alloy (RA) is complicated because the alloy tends to segregate into inhomogeneous mixtures of binaries and ternaries, arising from a large thermodynamic miscibility gap.^33^ Almost all the work reported on Al_*x*_In_1–*x*_As_*y*_Sb_1–*y*_ as an APD material has therefore involved a digital alloy (hereafter DA) growth method within the miscibility gap using a shutter sequence of AlSb, AlAs, AlSb, InSb, InAs, and Sb as described by Maddox et al.^19^

The published reports on SACM structures with avalanching widths varying from 250 to 1000 nm^23,27,31,32^ appear to suggest that the excess noise is not dependent on the avalanching region width, contrary to results observed in other material systems such as InP,^34^ InAlAs,^35^ and Al_0.85_Ga_0.15_As_0.56_Sb_0.44_ on InP.^13^ As the multiplication region thickness decreases, the electric fields necessary for multiplication will increase, resulting in a smaller difference between *a* and *b* (i.e., a larger *k* value) and thus higher excess noise. Despite the very low noise properties reported in the Al_*x*_In_1–*x*_As_*y*_Sb_1–*y*_ system, a systematic study of the impact of different multiplication region widths has yet to be undertaken.

In this work, we undertake such a systematic investigation into the avalanche multiplication and excess noise of four Al_0.7_In_0.3_As_0.31_Sb_0.69_ p–i–n diodes with avalanching widths varying from 103 to 1511 nm (henceforth referred to as P1–4) and an SACM APD (henceforth referred to as SACM1) using an Al_0.3_In_0.7_As_0.64_Sb_0.36_ absorption layer capable of detection wavelength up to 2.1 μm at room temperature with a 500 nm thick Al_0.7_In_0.3_As_0.31_Sb_0.69_ multiplication layer. These measurements enable the ionization coefficients and excess noise in Al_0.7_In_0.3_As_0.31_Sb_0.69_ to be extracted over a much wider electric field range than that hitherto investigated. The results show that the excess noise decreases rapidly from *F*_e_ = 6 to 2.6 @ *M*_e_ = 20 as the avalanche region width increases from 103 to 1511 nm, similar to the behavior seen in Al­(Ga)­AsSb on InP multiplication regions.^13,36^ Furthermore, the multiplication and excess noise results from SACM1 are in good agreement with those seen in a similar thickness p–i–n diode.

## Experimental Results

### Homojunction p–i–ns

In order to understand the multiplication and excess noise behavior in structures with different avalanching widths, four Al_0.7_In_0.3_As_0.31_Sb_0.69_ homojunction p–i–ns with nominal *i*-region thicknesses of *i* = 103, 509, 941, and 1511 nm were grown on GaSb using solid source molecular beam epitaxy. Due to the well-documented difficulty in growing Al_0.7_In_0.3_As_0.31_Sb_0.69_ as a random alloy,^19^ the growth in this study was also done as a digital alloy (DA) using a largely similar scheme of alternating layers of AlSb, AlAs AlSb, Sb, In, InAs, In, and Sb as detailed in the Supporting Information Section S1. The structure details for all four p–i–ns are listed in Table S3.1. The p^+^ and n^+^ doping were done with Be and Si, respectively, with levels of ∼2 × 10^18^ cm^–3^. The XRD ω–2θ scans shown in Supporting Information Section S2 demonstrate that good lattice matching can be obtained with this growth technique. The p^+^ Al_0.7_In_0.3_As_0.31_Sb_0.69_ cladding layer was kept to 300 nm in all except the 941 nm thick p–i–n, which had a thinner 100 nm p^+^ cladding layer. Different diameter mesa diodes with optical windows were fabricated using wet chemical etching (as detailed in Supporting Information Section 1). Dark current–voltage (*I*–*V*), capacitance–voltage (*C*–*V*), avalanche multiplication, and excess noise measurements were undertaken.

Our p–i–ns show negligible series resistance from forward *I*–*V* as shown in Figure 1a. The high reverse dark *I*–*V* seen here showed perimeter scaling, suggesting that the dark current is primarily dominated by surface leakage. The *i*-region thickness and doping levels determined from a 1D Poisson model fitting to these *C*–*V* measurements with a dielectric constant of 12.3 are shown by the black lines in Figure 1b and given in Table 1. The accuracy of the *i*-region width was confirmed by secondary ion mass spectroscopy (SIMS) measurements on these layers as shown in Table 1 and described in Supporting Information Section 4.

**Figure 1:**
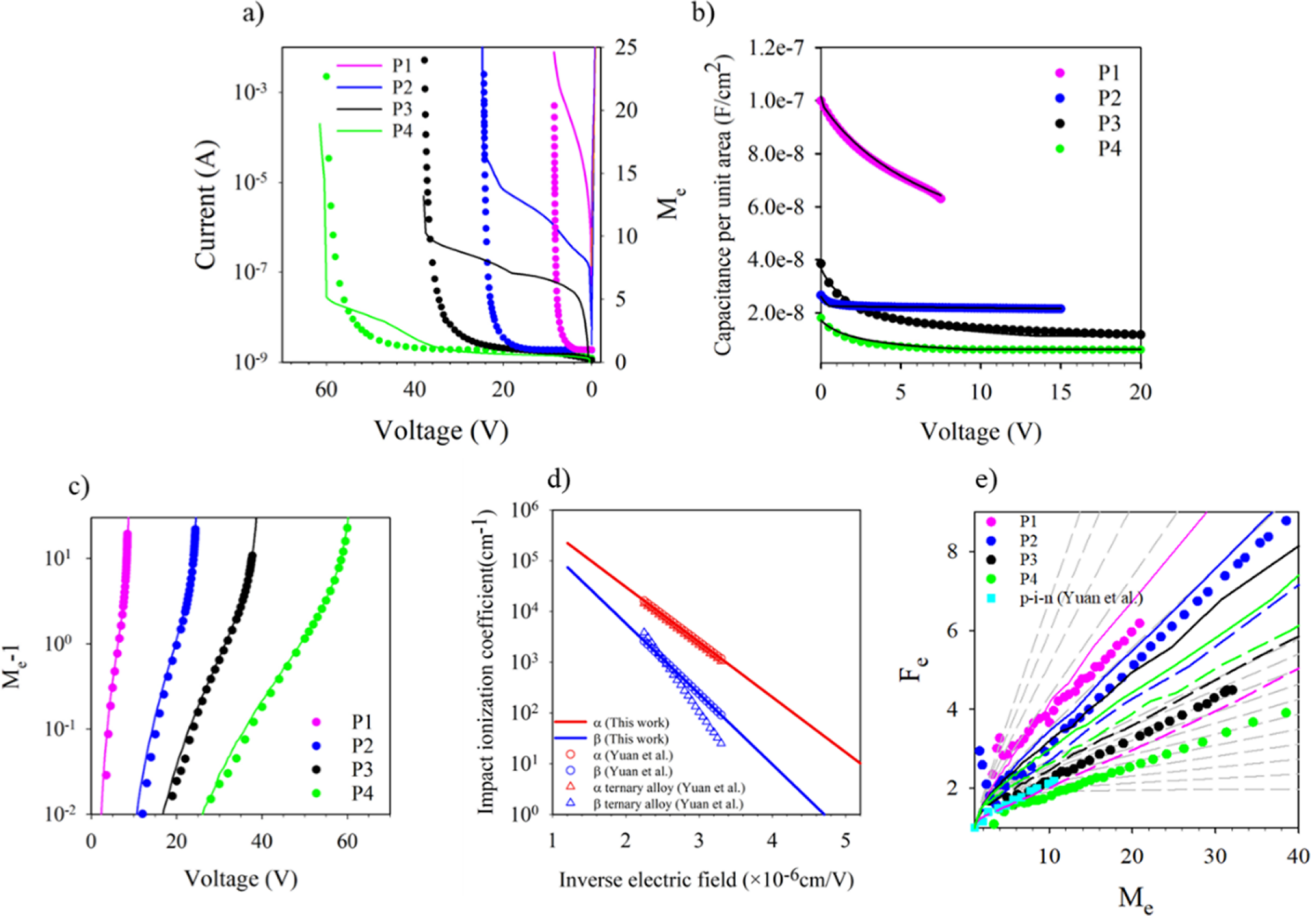
(a) Dark currents and *M*_e_ for P1–P4. (b) *C*–*V* measurements on P1–P4 (symbols) and the 1-D Poisson model fitting (black lines). (c) *M*_e_ – 1 on a log plot for P1–P4 (symbols) versus reverse bias. Colored lines are simulated results from the RPL model. (d) Ionization coefficients as determined from multiplication measurements on P1–P4 (lines) plotted with data from Yuan et al.^29^ (symbols). (e) *F*_e_ versus *M*_e_ for P1–P4 (circles). Result from Yuan et al.^29^ for 1000 nm p–i–n (cyan square). Gray dashed lines are local McIntyre lines from *k* = 0–0.1 in steps of 0.01 and *k* = 0.1–0.6 in steps of 0.1. Solid color lines are local model and dash lines are nonlocal model with *E*_the(h)_ = 2 eV.

**Table 1:** Structure Details for Four p–i–ns

layer no.	Nominal *i*-region thickness (nm)	*C*–*V* fitted region thickness (nm)	SIMS *i*-region thickness (nm)	*C*–*V* fitted *i*-region doping (×10^15^ cm^–3^)
P1	103	99	97	30
P2	509	482	508	11
P3	941	950	950	22
P4	1511	1439	1457	7

The pure electron-initiated multiplication (*M*_e_) of these four p–i–ns was measured using a 530 nm wavelength for P1, P2, and P4 and a 455 nm wavelength for P3 (due to its thinner p^+^ cladding layer thickness). The absorption coefficient in Al_0.7_In_0.3_As_0.31_Sb_0.69_ is 2.4 × 10^5^ cm^–1^ and 1 × 10^5^ cm^–1^ at a wavelength of 455 and 530 nm, respectively,^29^ which ensures more than 90% of the light is absorbed in the top cladding layers. Initial measurements showed that the *M*_e_ obtained was extremely sensitive to any scattered light falling on the mesa sidewalls and mesa floor, resulting in significant hole-initiated multiplication, also contributing to the multiplication obtained. This is described in more detail in Section S5. To solve this problem, a metal blocking layer was deposited around the mesa so that only light falling on the top central optical window contributes to the photocurrent (shown in Figure S5). The *M*_e_ was determined from the reverse bias-dependent photocurrent after accounting for the increasing primary photocurrent due to the depletion edge moving in the cladding layers.^37^ The avalanche multiplication breakdown voltage for all four p–i–ns agrees well with reverse *I*–*V* measurements, as shown in Figure 1a. Hole-initiated multiplication (*M*_h_) was also estimated by deliberately illuminating the mesa floor of devices from P2 and P4 without the metal blocking layer as described in Supporting Information Section S6.

These multiplication results are also plotted as a log­(*M*_e_ – 1) in Figure 1c to show the full range of multiplication obtained. With knowledge of the electric field profiles from *C*–*V* and SIMS measurements and the multiplication characteristics from these four layers, the impact ionization coefficients for both electrons and holes were determined using a numerical random path length (RPL)^38^ model. This model takes into consideration any varying electric field profile and the depletion into the cladding regions but ignores any “dead-space” effects.^39^ The ionization coefficients extracted in this manner are shown in Figure 1d together with published data from Yuan et al.^29^ but cover a much wider electric field range of 195–830 kV/cm. These ionization coefficients are capable of reproducing the measured *M*_e_ very well over 4 orders of magnitude as shown by the solid lines in Figure 1c. The parametrized equations are effectively identical to those given by Yuan et al.^29^ and are listed below in eqs 2 and 3

2 $$\alpha \left(E\right)=4.5\times 10^{6}\times \exp\left(-2.5\times 10^{6}/E\right)$$

3 $$\beta \left(E\right)=3.5\times 10^{6}\times \exp\left(-3.2\times 10^{6}/E\right)$$

validfor195kV/cm<E<830kV/cm

Figure 1e shows the excess noise from P1–P4 obtained using the circuit developed by Lau et al.^40^ The excess noise measurements were measured up to a multiplication of at least *M*_e_ = 20 and up to *M*_e_ = 40 in P2 and P4 as shown in Figure 1e. The *F*_e_ increases significantly from 2.6 to 6 at *M*_e_ = 20 as the multiplication width decreases from 1511 nm (P4) to 103 nm (P1) due to the increasing *k* as the operating electric field increases. The excess noise was measured up to *M*_e_ = 40 compared to *M*_e_ = 15 as reported by other groups. Figure 1e also shows the excess noise for a 1000 nm thick p–i–n reported by Yuan et al.,^29^ and this shows very similar results compared with our nominal 941 nm p–i–n device (P3). Previously, Jones et al. reported very low excess noise in an Al_*x*_In_1–*x*_As_*y*_Sb_1–*y*_ SACM;^23^ however, our results show much higher noise in P2, which has similar multiplication region width. Therefore, we have grown an SACM APD with a very similar structure to Jones et al.^23^ to investigate the validity of our excess noise in both p–i–ns and SACM APDs.

### Characterization of SACM APD

A schematic of SACM1 is shown in Figure 2a. A heavily doped n-type Al_0.7_In_0.3_As_0.31_Sb_0.69_ cladding layer is initially grown on an n-type GaSb substrate and buffer. This is followed by an undoped 500 nm thick Al_0.7_In_0.3_As_0.31_Sb_0.69_ multiplication region, a nominal 90 nm thick p-type Al_0.7_In_0.3_As_0.31_Sb_0.69_ charge sheet layer with a doping density of 1.1 × 10^17^ cm^–3^, a 0.6 × 10^17^ cm^–3^ 200 nm thick Al_*x*_In_1–*x*_As_*y*_Sb_1–*y*_ grading layer in which the Al composition is graded from *x* = 0.7 to 0.3, the undoped 1000 nm thick Al_0.3_In_0.7_As_0.64_Sb_0.36_ absorption region, and then heavily p-doped Al_0.7_In_0.3_As_0.31_Sb_0.69_. Finally, thin, heavily p-doped GaSb acts as the top contact layer. Beryllium (Be) was used as the p-type dopant, and tellurium (Te) was used as the n-type dopant. The material quality was investigated using high-resolution X-ray diffraction (HRXRD) and atomic force microscopy (AFM). In Figure 2b, the satellite peaks in the HRXRD rocking curves show superlattice periods of 3.192, 3.126, and 3.275 nm for the Al_0.7_In_0.3_As_0.31_Sb_0.69_ DA, Al_*x*_In_1–*x*_AsSb grading layer, and Al_0.3_In_0.7_As_0.64_Sb_0.36_ DA, with lattice mismatches of 14 arcsec, −142 arcsec, and 230 arcsec, respectively. The full width at half-maximum of the DA in each region is 44.8, 26.6, and 26.3 arcsec, indicating that the material has high quality at the interfaces. The AFM results showed a good surface morphology with clear atomic steps and a small rms roughness of 1.78 Å over a 20 μm × 20 μm area as shown in Figure 2c.

**Figure 2:**
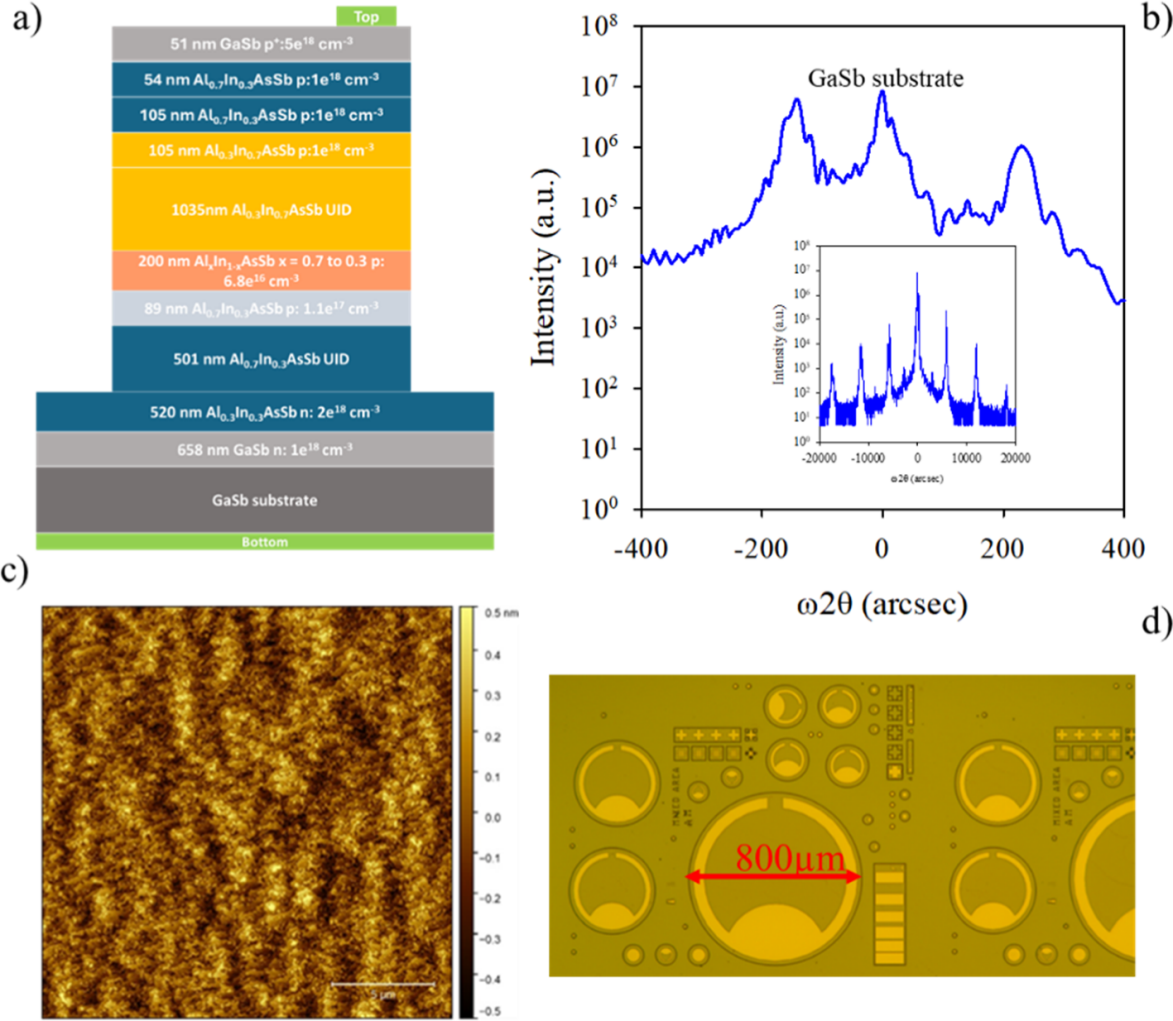
(a) Structure of the Al_0.3_In_0.7_As_0.64_Sb_0.36_/Al_0.7_In_0.3_As_0.31_Sb_0.69_ SACM APD grown by molecular beam epitaxy. (b) High-resolution X-ray diffraction measurements on the SACM APD layer. (c) AFM measurements on the SACM APD layer. (d) Microscope image of the fabricated devices.

Figure 3 shows a schematic diagram of the SACM APD structure and SIMS of SACM1. SIMS can easily differentiate between different regions of Al with 70%, 30%, and 0%. It also shows clearly how the p-type and n-type doping are changing across the structure. The SIMS results help validate the accuracy of the structure as it was grown.

**Figure 3:**
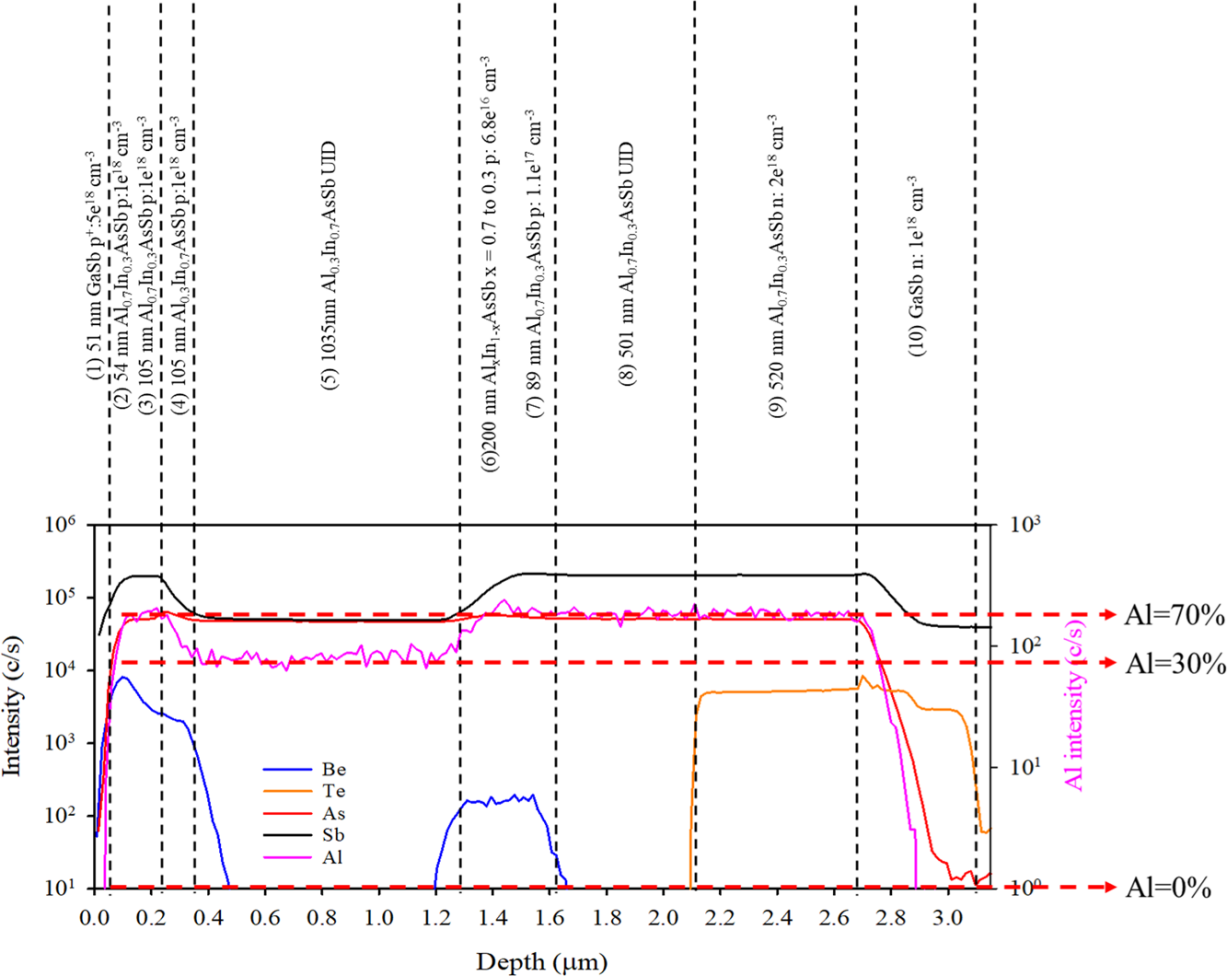
Confirmation of the SACM APD structure by SIMS.

Figure 4a shows the dark current density of SACM1 devices with device diameters of 200 and 400 μm under reverse-biased conditions. The increase in dark current at 23 V suggests depletion of the charge sheet and punch-through of the electric field into the absorption region. A repeatable sharp breakdown was observed at 42 V. The dark current scales with the area more than the perimeter of the devices after punch-through, indicating that this is mainly due to carriers from the absorption region crossing the charge barrier. The blue and red symbols show an example of the measured photocurrent when the optical window is illuminated by a 1.45 μm wavelength light using a phase-sensitive technique. No photocurrent signal is detected before punch-through as the carriers cannot overcome the potential barrier and enter the multiplication region. At a bias of ∼23 V, we observe the photocurrent signal, which increases with reverse bias because of impact ionization. In Figure 4b, the capacitance gradually decreases with increasing reverse bias and drops suddenly at a bias of 23 V, which indicates the punch-through of the electric field into the low-doped absorption region, agreeing well with the *I*–*V* measurements shown in Figure 4a. The *C*–*V* was simulated to investigate the doping density of SACM1. The simulation results, shown by the red solid line in Figure 4b, suggests that the absorber, grading layer, charge sheet, and multiplication region thickness are 900, 230, 90, and 535 nm, respectively, in close agreement with the structure design. Figure 4c shows the background doping as a function of depletion width with static dielectric constants of 14.2 and 12.3 for Al_0.3_In_0.7_As_0.64_Sb_0.36_ and Al_0.7_In_0.3_As_0.31_Sb_0.69_, respectively. The peak around 500 nm represents the charge sheet and grading layer doping in SACM1, and the doping peak value is found to be ∼1 × 17 cm^–3^. The total amount of charge, including the grading layer, was calculated to be 2.34 × 10^12^ cm^–2^, very close to the design value of 2.2 × 10^12^ cm^–2^. Figure 4d shows the simulation of the electric field profile at reverse biases of 23, 38, and 41 V, using the doping profile extracted from the *C*–*V* measurements.

**Figure 4:**
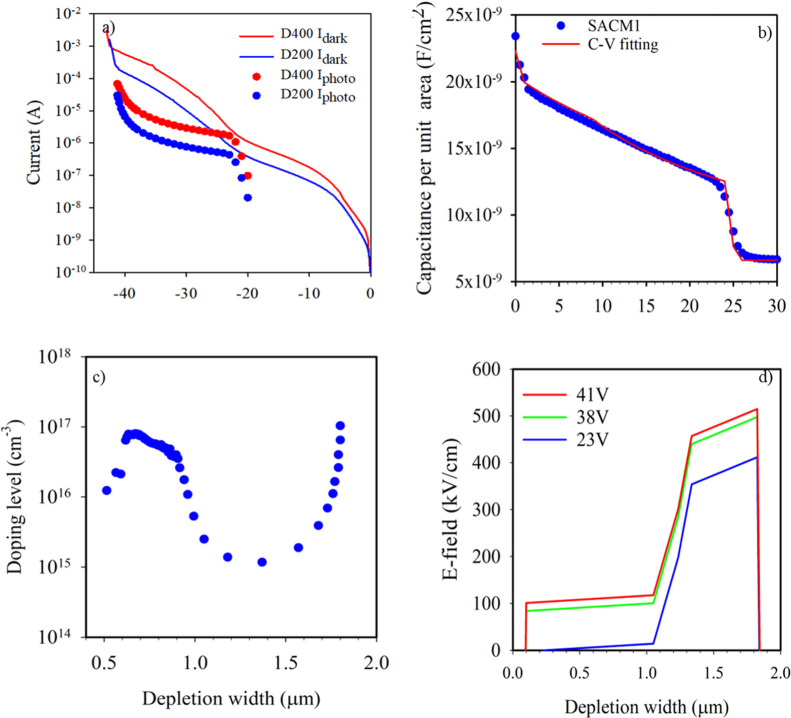
(a) Dark current (lines) and photocurrents (symbols) under illumination of an LED light at a wavelength of 1.45 μm at room temperature (b). *C*–*V* measurements (symbols) and best fit (red line) by solving a 1-D Poisson model. (c) Calculated doping density profile in SACM1 from measured *C*–*V*. (d) Electric field distribution for SACM1 at different reverse biases.

The external quantum efficiency (EQE) of SACM1 as a function of wavelength was determined using a tungsten halogen bulb and monochromator combination, as shown in Figure 5a. The primary EQE at 2 μm was measured to be 7.14% at *M*_e_ = 1 without any antireflection coating, and this EQE can be increased to 1406% by applying a higher reverse bias when *M*_e_ = 197. This value of *M*_e_ is larger than the 150 reported in Jones et al.^23^ Figure 5b shows the avalanche multiplication at different reverse biases for SACM1 when illuminated by light at wavelengths of 1.45 μm (blue circle) and 2 μm (pink circle). The avalanche multiplication of SACM1 shows wavelength independence due to “pure” electron injection up to *M*_e_ = 197. Measurements undertaken on several devices with different optical powers and wavelengths up to 2 μm also gave similar multiplication characteristics. The RPL model^38^ was used to simulate the SACM1’s multiplication with the electric field distribution profile obtained from the *C*–V measurement and impact ionization coefficients shown in Figure 1d. The avalanche multiplication in SACM1 is determined to be *M*_e_ = 1.4 at the punch-through voltage of 23 V by fitting the results to RPL model simulation, and the simulated avalanche multiplication from 23 V onward agrees well up to a *M*_e_ = 197 with the measured multiplication results. In SACM1, the *F*_e_ (blue circles) was measured up to *M* = 30 and yielded an excess noise factor of *F*_e_ = 5 at *M*_e_ = 20, the same as that for P2, which has a similar multiplication region thickness as shown in Figure 5c (green circles). This is expected since the impact ionization will happen only in the high-field multiplication region with negligible multiplication in the low electric field absorber region.^7,14,15^

**Figure 5:**
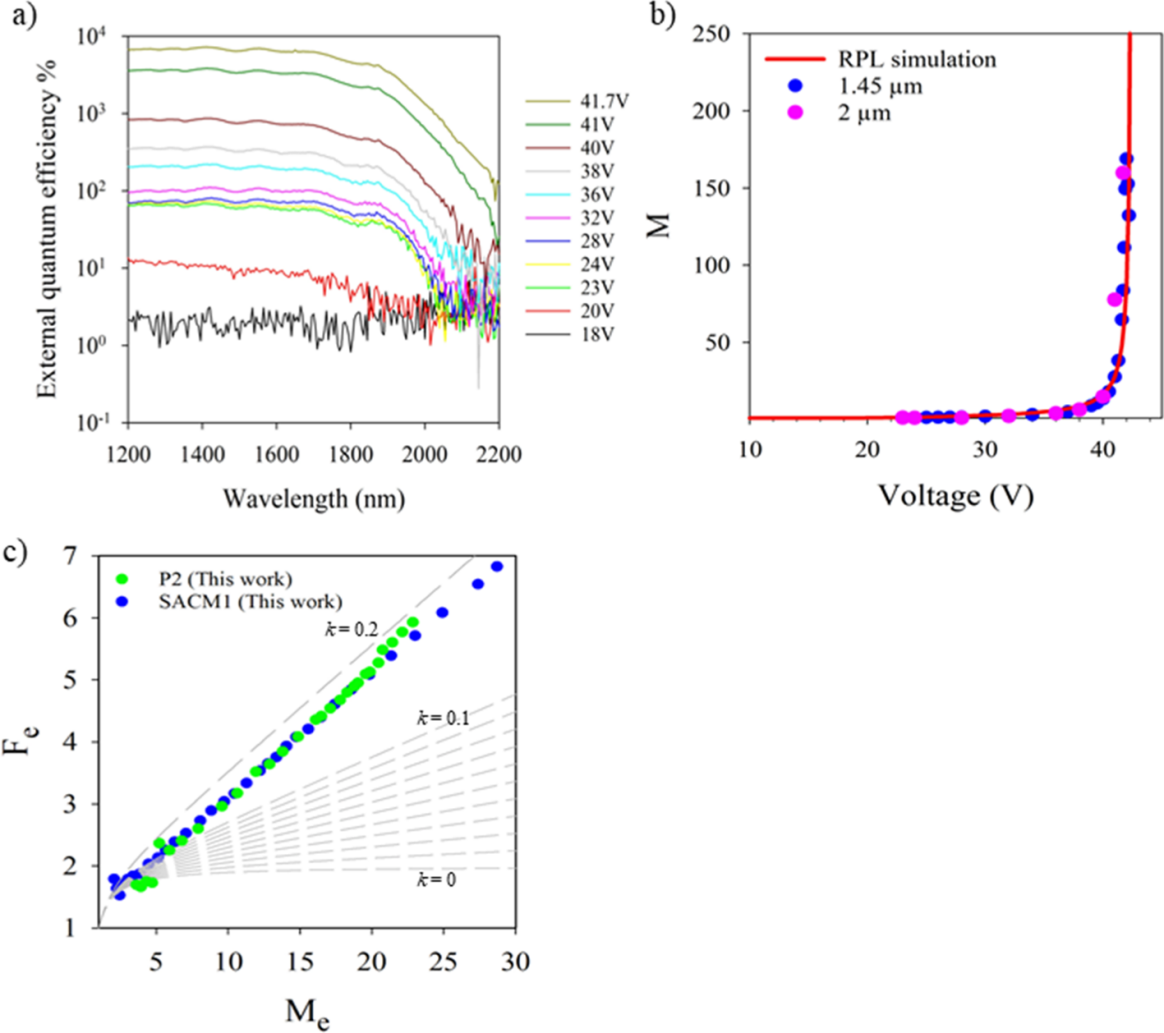
(a) External quantum efficiency of SACM1 at different reverse biases. (b) Avalanche multiplication of SACM1 at a wavelength of 1.45 μm (blue) and 2 μm (pink); the red solid line is the RPL model^38^ simulation. (c) Excess noise of SACM1 compared with that of P2. Gray dashed lines are local McIntyre lines from *k* = 0 to 0.1 in steps of 0.01 and *k* = 0.1 to 0.2 in steps of 0.1.

## Discussion

Figure 6a compares the ionization coefficients of Al_0.7_In_0.3_As_0.31_Sb_0.69_ (from Figure 1d) with the ionization coefficients of Al_0.85_Ga_0.15_As_0.56_Sb_0.44_,^41^ Al_0.55_Ga_0.45_As_0.56_Sb_0.44,_ ^16^ and InAlAs,^42^ all grown on InP. The β/α ratio of Al_0.7_In_0.3_As_0.31_Sb_0.69_ is clearly larger than that of Al_0.85_Ga_0.15_As_0.56_Sb_0.44_ ^41^ and appears broadly similar to that of Al_0.55_Ga_0.45_As_0.56_Sb_0.44_ ^16^ and InAlAs.^42^ If we compare the *F*_e_ vs *M*_e_ results of P4 with that of a 1550 nm thick Al_0.55_Ga_0.45_As_0.56_Sb_0.44_ p–i–n^16^ on InP in Figure 6b, we can see that they are very similar, as are the β/α ratios in the two materials. P3 with a thinner multiplication region of ∼1000 nm will operate at a higher electric field and so effectively has a larger *k*, giving rise to higher *F*_e_. This P3 excess noise result is also in very good agreement with the excess noise results of the similar 1000 nm thick p–i–ns reported by Yuan et al.^30^ (down triangles) as shown in Figure 6b but the results of Woodson et al.^26^ (purple circle) are surprisingly low for an 890 nm thick p–i–n. The *F*_e_ vs *M*_e_ results of the 1000 nm InAlAs p–i–n^35^ are, however, much larger despite having what looks like a similar *k*. Goh et al.^35^ reported that the excess noise in InAlAs could be replicated by the RPL model if ionization threshold energies of 3.2 eV for electrons and 3.5 eV for holes were used. The 1020 nm thick Al_0.85_Ga_0.15_As_0.56_Sb_0.44_ on InP has a much smaller *k* ^41^ and consequently a much lower *F*_e_ vs *M*_e_.^13^

**Figure 6:**
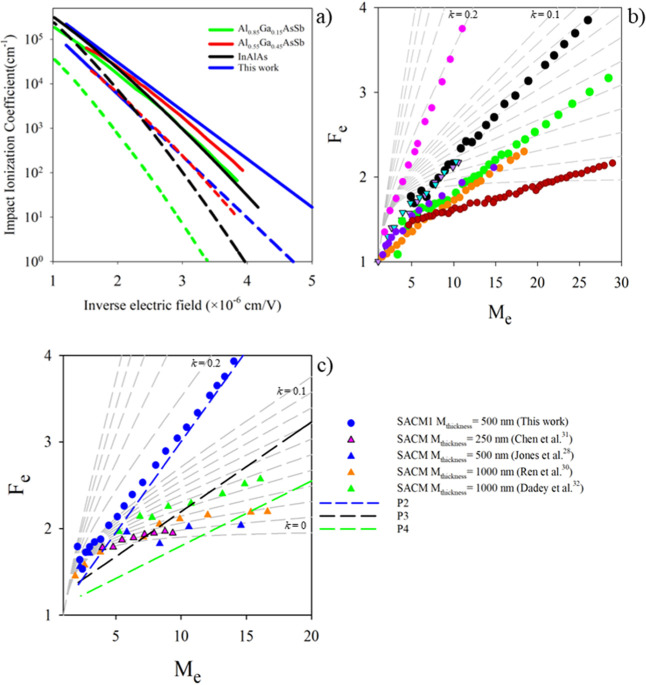
(a) *a* (solid lines) and *b* (dashed lines) for Al_0.7_In_0.3_As_0.31_Sb_0.69_ (blue) compared to those of Al_0.85_Ga_0.15_As_0.56_Sb_0.44_ ^41^ (green), Al_0.55_Ga_0.45_As_0.56_Sb_0.44_ ^16^ (red), and InAlAs.^42^ (b) Excess noise of p–i–ns P3 (black circles) and P4 (green circles) compared to Yuan et al. (down triangles)^30^ and Woodson et al. (purple circle).^26^ Also shown are the noise results of 1500 nm p–i–ns of Al_0.55_Ga_0.45_As_0.56_Sb_0.44_ (orange circles)^16^ and 1000 nm Al_0.85_Ga_0.15_As_0.56_Sb_0.44_ (blue circles)^13^ and a 1000 nm InAlAs p–i–n (pink circles),^35^ all grown on InP. (c) Al_*x*_In_1–*x*_As_γ_Sb_1‑__γ_-based SACM APD results from the literature (triangles) compared with results for the SACM APD reported here (blue circle).^22,27,31,32^ The noise results for P2, P3, and P4 are shown as colored dashed lines (blue, black, and green, respectively). Gray dashed lines are local McIntyre lines from *k* = 0 to 0.1 in steps of 0.01 and *k* = 0.1 to 0.6 in steps of 0.1.

However, this excess noise cannot be predicted by the local model as shown in Figure 1e (solid colored lines), and attempts to implement a hard threshold energy of 2 eV for both electrons and holes in Al_0.7_In_0.3_As_0.31_Sb_0.69_ using the ionization coefficients from eqs 2 and 3 manage to reduce the predicted noise (dashed color lines) from that of the local model but still gives a poor fit to the experimental results. Ong et al. showed that the only way to explain this very low noise behavior in the Al_0.85_Ga_0.15_As_0.56_Sb_0.44_ alloy system was by modifying the conventional exponential shape of the ionization probability density function (PDF) using a Weibull–Fréchet (WF) distribution for the ionization.^13^ The excess noise obtained in P3 and P4 appears to be lower than that which could be accounted for by the *k* seen from the ionization coefficients, even with relatively large threshold energies, suggesting that the ionization process may have a similar WF-PDF due to the presence of Sb. Finally, the excess noise from several publications on SACM APDs with multiplication region widths varying from 250 to 1000 nm^22,27,31,32^ is shown in Figure 6c. While the noise measurement technique used in our work^40^ enables the measurements of *F*_e_ at *M*_e_ up to 30 for P3 and >40 for P2 and P4, the results of the SACM APDs from the literature in Figure 6c cover a limited range up to a *M*_e_ = 16. These results do not appear to show the increase in noise we might expect to see as the multiplication region width decreases and *k* increases. Our SACM1 has a similar APD structure as reported previously by Jones et al.;^23^ however, our noise is much higher than their work but agrees with P2, which has a similar multiplication region width. The reason for this is unclear at present, but one of these noise results^32^ was taken at low temperature and this can affect the *F*_e_ vs *M*_e_ that is obtained. The *F*_e_ values in all these structures (and P4) appear to have values between 1.8 and 2.3 at *M*_e_ = 15. This level of excess noise is only reached at much higher *M* values in a 1000 nm thick Al_0.85_Ga_0.15_As_0.56_Sb_0.44_/GaAsSb SACM APD.^14^ For near-room-temperature operation of a high-sensitivity extended SWIR detector, it may be that a T2SL absorber and Al_0.85_Ga_0.15_As_0.56_Sb_0.44_ grown on InP can be a viable alternative to GaSb-based alloys, depending on other factors such as the wavelength of operation, speed, and operating voltage.

## Conclusion

Based on a study of four Al_0.7_In_0.3_As_0.31_Sb_0.69_ p–i–n diodes with avalanching widths varying from 103 to 1511 nm, we find that the excess noise varies from *F*_e_ = 2.6 to 6 at *M*_e_ = 20 as the multiplication region width decreases. The impact ionization coefficients as determined over a large electric field range from 190 kV/cm to 830 kV/cm are capable of replicating the multiplication reasonably accurately for a wide range of avalanching widths. An SACM APD capable of detection up to 2 μm is demonstrated with a maximum *M*_e_ of 197 and *F*_e_ = 5 at *M*_e_ = 20.

## Supplementary Material



## Notes

Details and fabrication details; X-ray results of P1 to P4; structure details of P1 to P4 and SACM 1; SIMS of P1 to P4; SU-8 and metal blocking layer p–i–n device cross section; and effect of multiplication on pure and mixed injection when the metal blocking of side illumination is used (PDF)
