# Supplementary material for: A Study of the Avalanche Multiplication and Excess Noise in Al x In1–x AsγSb1‑ γ Avalanche Photodiodes Lattice-Matched to GaSb
Source: ACS Photonics. 2026 Feb 13;13(5):1275–83. doi: 10.1021/acsphotonics.5c02166 (PMC12964527; doi:10.1021/acsphotonics.5c02166)
Supplement: Supplementary file 1 [file ph5c02166_si_001.pdf]

A study of the avalanche multiplication and excess noise in $Al_xIn_{1-x}As_\gamma Sb_{1-\gamma}$ avalanche photodiodes lattice matched to GaSb

Xiao Jin[1,‡], Wenguang Zhou[2,3,4,‡], Yang Zhao[1,‡], Qingyu Tian[1,‡], Xin Yi[5], Xiaofeng Tao[1], Adam Craig,[6] Mrudul Modak,[5] Andrew Marshall,[6] Yingqiang Xu,[2,3] Guowei Wang,[2,3,*] John P. R. David,[1] & Gerald S. Buller,[5]*

[1]*Department of Electronics and Electrical Engineering, University of Sheffield, Sheffield, S1 3JD, UK*

[2]*Key Laboratory of Optoelectronic Materials and Devices， Institute of Semiconductors, Chinese Academy of Sciences, Beijing 100083, China*

[3]*Center of Materials Science and Optoelectronics Engineering，University of Chinese Academy of Sciences, Beijing 100049, China*

[4]*China Electric Power Research Institute, Beijing,102209, China*

[5]*Institute of Photonics and Quantum Sciences, School of Engineering and Physical Sciences, Heriot-Watt University, Edinburgh, EH14 4AS, UK*

[6]*Department of Physics, University of Lancaster, Lancaster, LA1 4WA, UK*

xin.yi@hw.ac.uk & wangguowei@semi.ac.cn

Supplementary Section 1: Growth Details and fabrication details

The devices were grown on n-type GaSb (001) substrates using a Veeco Gen II Molecular Beam Epitaxy (MBE) system equipped with group III SUMO® cells and group V valved crackers using As_2 and Sb_2. The substrate temperature was held at 430°C calibrated by the GaSb (5× to 3×) substrate reconstruction transition temperature (T_c). In-situ reflection high-energy electron diffraction (RHEED) was used to calibrate the growth rates of In and Al which were found to be 0.4 ML/s. The $Al_xIn_{1-x}As_ySb_{1-y}$ materials were grown as a DA of the binary alloys, utilizing a DA period of ~3 nm (10 MLs) and a Migration-Enhanced Epitaxy (MEE) interface growth method [1] with the following layer sequence: AlSb, AlAs AlSb, Sb, In, InAs, In, Sb. The DA growth technique used in this work differs slightly from the previous work done by Jones *et al.* [2] in that the substrate temperature is reduced from 480°C to 430°C and the growth rate is also reduced from 0.75 ML/s to 0.4 ML/s. Complete growth details are provided elsewhere [1]. The lower growth rate and MEE interface growth method used here should enhance the crystal quality of the material by producing smoother interfaces and reducing point defects [3]. The circular mesa diode for all wafers was fabricated by standard photolithography and wet chemical etching.

Supplementary Section 2: X-ray results of P1 to P4

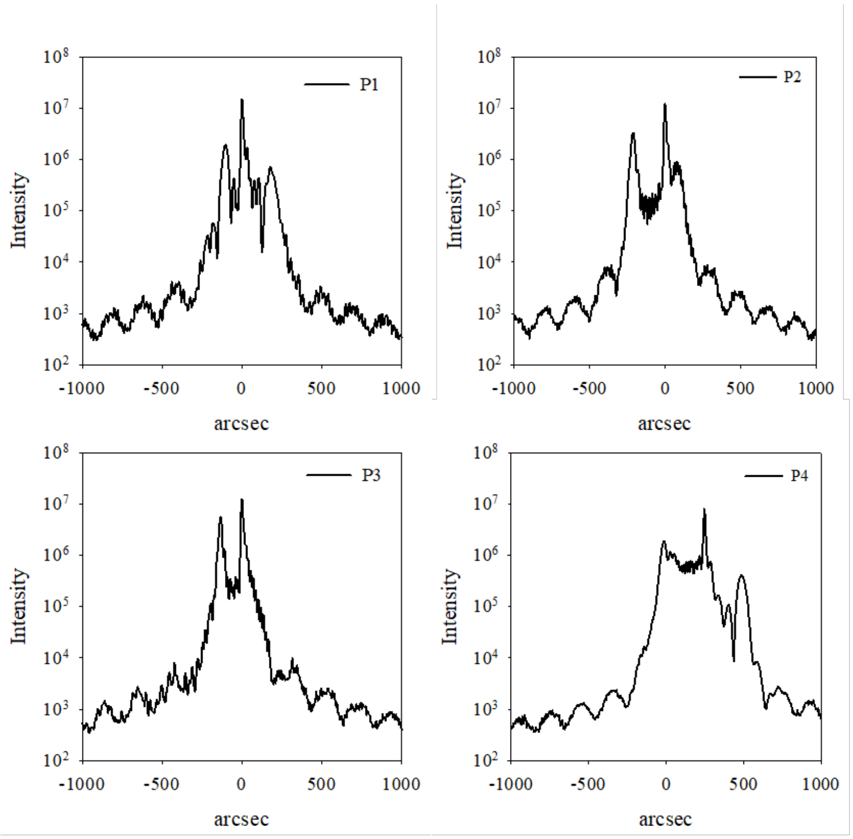

Fig. S2. a) X-ray results of layer P1. b) X-ray results of layer P2. c) X-ray results of layer P3. d) X-ray results of layer P4

$\omega - 2\theta$ x-ray scans were undertaken on all the layers and the results for the 4 p-i-n structures are shown below. The AlInAsSb epilayer peak was within 500 arc sec of the substrate, suggesting that near lattice matched conditions were achieved in all these cases using the growth technique described in Section 1 above.

Supplementary Section 3: Structure details of P1 to P4 and SACM 1

Table 3.1. Structure details of P1 to P4

Layer	Material	Doping(cm^{-3})	Thickness(nm)
PIN1	GaSb	p^{++}: 5×10^{18}	109
	$Al_{0.7}In_{0.3}As_{0.31}Sb_{0.69}$	p^{+}: 2×10^{18}	306
	$Al_{0.7}In_{0.3}As_{0.31}Sb_{0.69}$	UID	103
	$Al_{0.7}In_{0.3}As_{0.31}Sb_{0.69}$	n^{+}: 2×10^{18}	308
	GaSb	n^{+}: 1.7×10^{18}	439
	GaSb substrate		
PIN2	GaSb	p^{++}: 5×10^{18}	109
	$Al_{0.7}In_{0.3}As_{0.31}Sb_{0.69}$	p^{+}: 2×10^{18}	306
	$Al_{0.7}In_{0.3}As_{0.31}Sb_{0.69}$	UID	509
	$Al_{0.7}In_{0.3}As_{0.31}Sb_{0.69}$	n^{+}: 2×10^{18}	308
	GaSb	n^{+}: 1.7×10^{18}	439
	GaSb substrate		
PIN3	GaSb	p^{++}: 5×10^{18}	100
	$Al_{0.7}In_{0.3}As_{0.31}Sb_{0.69}$	p^{+}: 2×10^{18}	105
	$Al_{0.7}In_{0.3}As_{0.31}Sb_{0.69}$	UID	941
	$Al_{0.7}In_{0.3}As_{0.31}Sb_{0.69}$	n^{+}: 2×10^{18}	207
	GaSb	n^{+}: 1.7×10^{18}	549
	GaSb substrate		
PIN4	GaSb	p^{++}: 5×10^{18}	109
	$Al_{0.7}In_{0.3}As_{0.31}Sb_{0.69}$	p^{+}: 2e18	308
	$Al_{0.7}In_{0.3}As_{0.31}Sb_{0.69}$	UID	1511
	$Al_{0.7}In_{0.3}As_{0.31}Sb_{0.69}$	n^{+}: 2×10^{18}	308
	GaSb	n^{+}: 1.7×10^{18}	439
	GaSb substrate		

Table 3.2. Structure details of SACM1

Layer	Material	Doping(cm^{-3})	Thickness(nm)
SACM1	GaSb	p^{++}: 9×10^{18}	51
	$Al_{0.7}In_{0.3}As_{0.31}Sb_{0.69}$	p^{++}: 1×10^{19}	54
	$Al_{0.7}In_{0.3}As_{0.31}Sb_{0.69}$	p^{+}: 1×10^{18}	105
	$Al_{0.3}In_{0.7}As_{0.64}Sb_{0.36}$	p^{+}: 1×10^{18}	105
	$Al_{0.3}In_{0.7}As_{0.64}Sb_{0.36}$	UID	1035
	$Al_xIn_{1-x}AsSb$<br>x=0.7 to 0.3 Grading	p: 6.8×10^{16}	200
	$Al_{0.7}In_{0.3}As_{0.31}Sb_{0.69}$	p: 1.1×10^{17}	89
	$Al_{0.7}In_{0.3}As_{0.31}Sb_{0.69}$	UID	501
	$Al_{0.7}In_{0.3}As_{0.31}Sb_{0.69}$	n^{+}: 2×10^{18}	520
	GaSb	n^{+}: 1.4×10^{18}	658
	GaSb substrate		

Supplementary Section 4: Secondary-Ion Mass Spectrometry results (SIMS) of P1 to P4

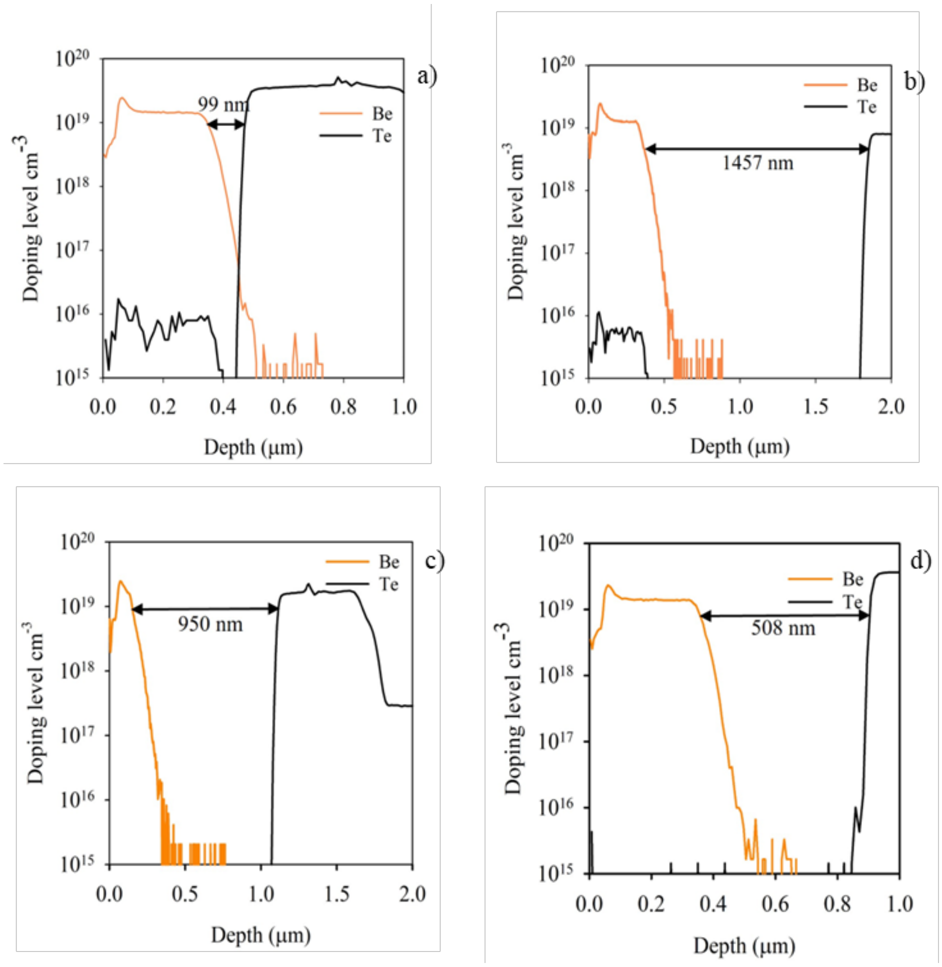

Fig. S4.a) SIMS results of P1. b) SIMS results of P2. c) SIMS results of P3. d) SIMS results of P4

Supplementary Section 5: SU-8 and metal blocking layer p-i-n device cross section

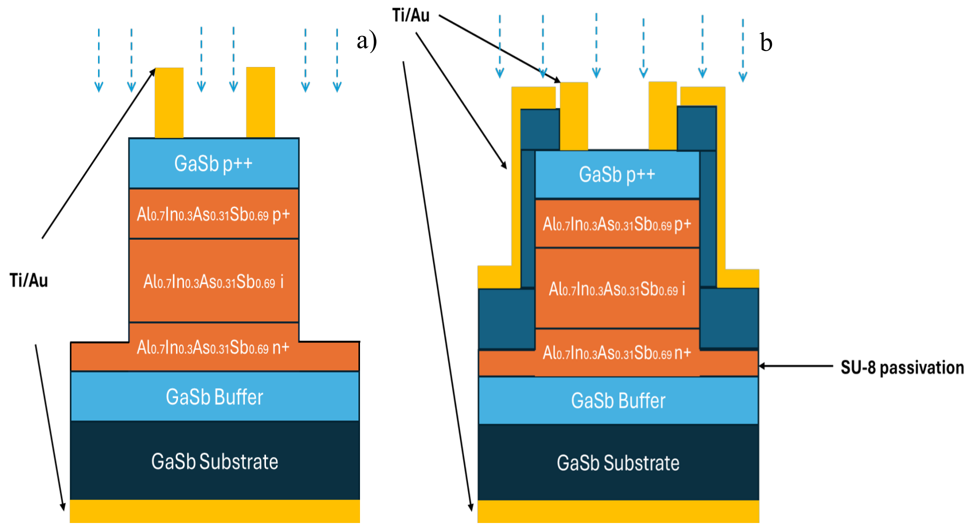

Fig.S5. a)A schematic diagram of the device structure without metal blocking layer. b) A schematic diagram of the device structure with metal blocking layer

Fig S5.a) shows the schematic cross section of a normal mesa diode. When the top optical window is illuminated by short wavelength light, the photocurrent that is measured depends on the absorption coefficient at that wavelength and also the electron minority carrier diffusion length (L_e). If L_e is short and there is also stray light falling on the sides/floor of the mesa, the photocurrent can be dominated by holes entering the high field depletion region. Fig. S5.b) shows how we use a metal blocking layer to prevent any light from outside the optical window region contributing to the photocurrent.

Supplementary Section 6: Effect of multiplication on pure and mixed injection when metal blocking of side illumination is used.

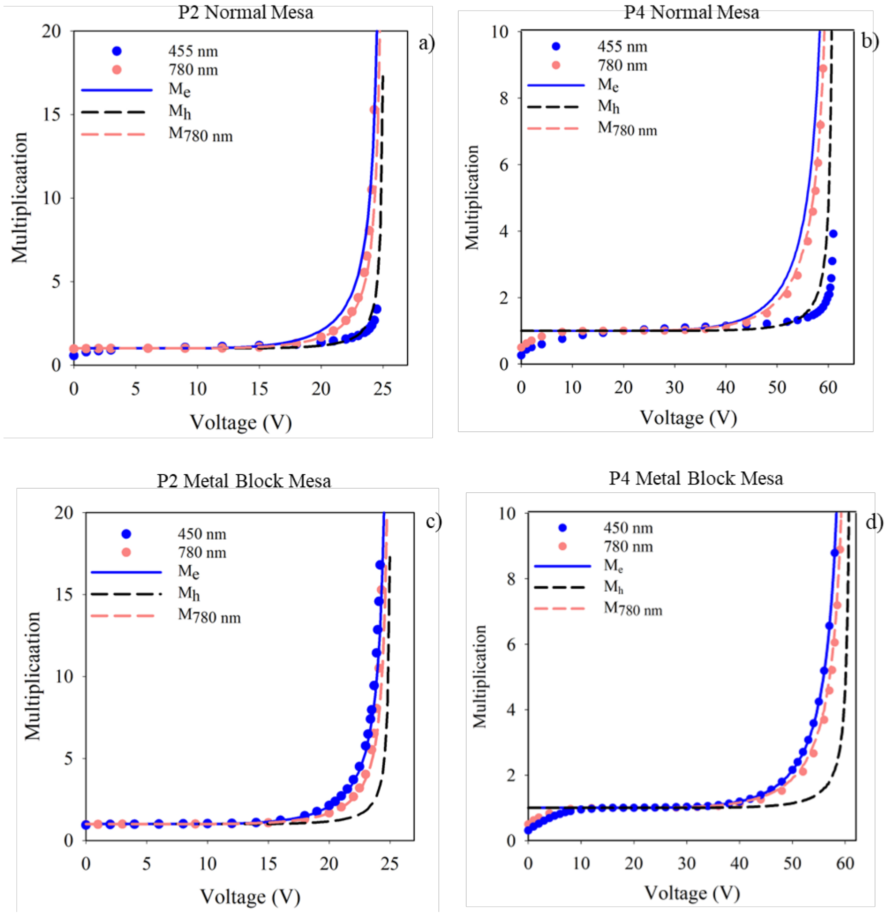

Fig. S6.a) Avalanche multiplication for P2 without metal block layer. b) Avalanche multiplication for P4 without metal block layer. c)Avalanche multiplication for P2 with metal block layer. d) Avalanche multiplication for P4 with metal block layer. Symbols are experimental data that illuminates the device on top of the device using 455 nm wavelength light (Blue) and 780 nm (pink). Blues solid lines, black dash lines and pink dashed lines are simulated M_e, M_h and M_{mix} using a random path length model(RPL) [4]and ionization coefficient from Yuan *et al.* [5]

Figs. S6.a) and Fig. S6.b) show that when 455nm illumination is used (blue symbols) on the top optical window of the normal mesa devices of P2 and P4, we see a multiplication that looks more like M_h than M_e. This suggests that carriers falling on the mesa sides and mesa floor dominate the photocurrent. When the mesa sides are protected by the metal blocking layer (Fig. S5.b), we see a M_e that agrees with the expected ionization coefficients given by equations (1) and (2) in the main paper (Figs.

S6.c and S6.d). The use of a longer wavelength illumination of 780nm gives rise to 'mixed' carrier multiplication due to electrons and holes created in the intrinsic region, and this is relatively less sensitive to the presence any illumination on the sides/mesa floor. In the figures, the solid blue and dashed black lines are the expected M_e and M_h calculated using the ionization coefficients for this material. The pink dashed line is the 'mixed' multiplication assuming that the 780 nm light has an absorption coefficient of 1.14×10^4 cm^{-1} and L_e is > 0.5 mm.

References

1. Y. Lyu, X. Han, Y. Sun, Z. Jiang, C. Guo, W. Xiang, Y. Dong, J. Cui, Y. Yao, D. Jiang, G. Wang, Y. Xu, and Z. Niu, "Digitally grown AlInAsSb for high gain separate absorption, grading, charge, and multiplication avalanche photodiodes," J. Cryst. Growth **482**, 70–74 (2018).

2. A. H. Jones, Y. Shen, K. Sun, D. Chen, S. D. March, S. R. Bank, and J. C. Campbell, "Room-temperature bandwidth of 2-μm AlInAsSb avalanche photodiodes," Opt. Express **29**, 38939 (2021).

3. D.-W. J. Wen-Guang Zhou Xiang-Jun Shang, Dong-Hai Wu, Fa-Ran Chang, Jun-Kai Jiang, Nong Li, Fang-Qi Lin, Wei-Qiang Chen, Hong-Yue Hao, Xue-Lu Liu, Ping-Heng Tan, Guo-Wei Wang, Ying-Qiang Xu, and Zhi-, "On the origin of carrier localization in AlInAsSb digital alloy," Chinese Phys. B **32**, 88501 (n.d.).

4. D. S. Ong, K. F. Li, G. J. Rees, J. P. R. David, and P. N. Robson, "A simple model to determine multiplication and noise in avalanche photodiodes," J. Appl. Phys. **83**, 3426–3428 (1998).

5. Y. Yuan, J. Zheng, A. K. Rockwell, S. D. March, S. R. Bank, and J. C. Campbell, "AlInAsSb Impact Ionization Coefficients," IEEE Photonics Technol. Lett. **31**, 315–318 (2019).
